# Functional and quality-of-life outcomes after metal-on-polyethylene articulating spacer implantation for periprosthetic knee infection: a retrospective evaluation of a prospectively collected cohort

**DOI:** 10.1007/s00402-026-06260-0

**Published:** 2026-04-17

**Authors:** Giovanni Balato, Enrico Festa, Tiziana Ascione, Domenico De Mauro, Donato Di Gennaro, Massimo Mariconda

**Affiliations:** 1 Department of Public Health, Orthopaedic unit, University of Naples Federico II, Naples, Italy; 2 Service of Infectious Diseases, Ospedale Antonio Cardarelli, Naples, Italy; 3 Department of Orthopedics, Ageing and Rheumatological Sciences, Agostino Gemelli University Polyclinic, Rome, Italy

**Keywords:** Periprosthetic Joint Infection (PJI), Articulating Spacer (metal-on-polyethylene), Quality of Life, Knee spacer, Functional Outcomes

## Abstract

**Introduction:**

This study aims to evaluate functional and quality-of-life (QoL) outcomes in patients with periprosthetic joint infection (PJI) undergoing implantation of a metal-on-polyethylene articulating knee spacer as part of a planned two-stage revision, and to explore preoperative predictors of meaningful postoperative improvement.

**Methods:**

This was a retrospective observational study including 108 consecutive patients with chronic PJI treated with a metal-on-polyethylene articulating spacer between 2016 and 2020 and followed prospectively for clinical outcomes. QoL and joint-specific function were assessed preoperatively and before planned reimplantation using EQ-5D-5 L, Western Ontario and McMaster University (WOMAC), and Knee Society Score (KSS). Minimum clinically important difference (MCID) thresholds were derived using a distribution-based method. Receiver operating characteristic (ROC) analysis identified baseline predictors of achieving MCID.

**Results:**

All outcome measures improved significantly after spacer implantation (*p* < 0.001), exceeding MCID thresholds for pain and function. The largest EQ-5D-5 L domain gain was for pain/discomfort. Predictive thresholds for achieving MCID were an EQ-5D-5 L index ≤ 0.44 and WOMAC index ≥ 42.5. Female sex was associated with better preoperative and postoperative WOMAC and KSS scores.

**Conclusion:**

Metal-on-polyethylene articulating spacers are associated with short-term improvements in pain, mobility, and QoL during the interval between stages of revision for PJI. Patients with poor baseline status may experience the greatest benefit, thereby supporting consideration of avoiding definitive reimplantation (1.5 stage). While these interim results may inform patient selection for leaving the spacer indefinitely, long-term follow-up is required to determine durability.

**Supplementary Information:**

The online version contains supplementary material available at 10.1007/s00402-026-06260-0.

## Introduction

Periprosthetic joint infection (PJI) is a severe complication of primary total knee arthroplasty (TKA) with a frequency of up to 1–2% [1, 2]. The two-stage exchange technique represents the standard treatment approach, with an 83 to 91% eradication rate [3]. However, this revision strategy has a considerable impact on the quality of life (QoL), and patients undergoing two-stage revision surgery experience extended periods of disability and social isolation [4], with possible development of depressive symptoms [5,6]. Although articulated cement-on-cement spacers have been shown to provide better function in the intermediate period and easier reimplantation than non-articulated spacers, patients often complain of pain and impaired knee function and QoL^7^. Metal-on-polyethylene spacers [8], due to better knee balance and alignment to cement-on-cement spacers, should theoretically provide better joint function and QoL, thus may serve in selected patients as a definitive solution within a 1.5-stage approach [9,10]. However, most published evidence [11,12] has focused on infection eradication rates, with limited prospective data on validated patient-reported outcome measures (PROMs) and predictors of postoperative improvement. This study aimed to evaluate QoL and function after metal-on-polyethylene spacer implantation in a consecutive cohort undergoing planned two-stage revision, and to identify baseline predictors of clinically meaningful improvement.

## Methods

### Patient selection

**Fig. 1:**
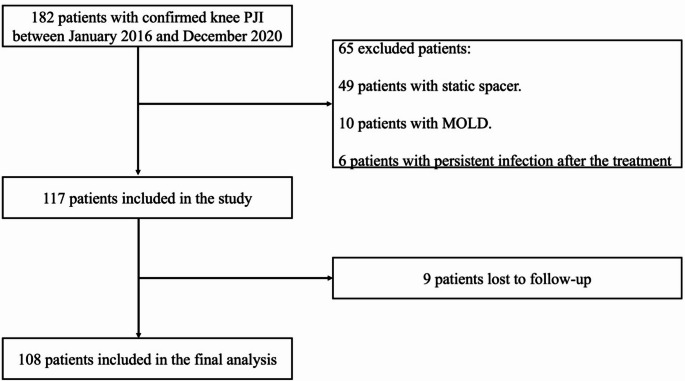
Flowchart of patient selection showing the inclusion and exclusion process leading to the final study cohort (*n* = 108). PJI, periprosthetic joint infection; MOLD, cement on cement spacer

This retrospective observational study based on a prospectively maintained institutional database included all patients diagnosed with chronic periprosthetic joint infection (PJI) of the knee who underwent two-stage exchange procedures at our institution between January 2016 and December 2020. The inclusion and exclusion criteria are reported in Table 1. As illustrated in Fig. 1 and 65 patients were excluded: 49 had a static spacer, 10 had a Cement-on-cement spacers (MOLD), and 6 had persistent infection after treatment. Of the 117 eligible patients, 9 were lost to follow-up, leaving 108 patients for final analysis. All the patients underwent implantation of a metal-on-polyethylene articulating spacer consisting of a new femoral component and a polyethylene tibial insert cemented with antibiotic-loaded bone cement, allowing joint articulation and early mobilization, according to a previously reported surgical technique [13].

**Table 1:** Inclusion and exclusion criteria

Inclusion criteria	Diagnosis of chronic PJI based on ICM 2018 criteria Age > 18 years Treatment with metal-on-polyethylene articulating spacer
Exclusion criteria	Chronic inflammatory joint disease Severe soft tissue coverage defect Local or systemic complications that prevented early full weight-bearing Intraoperative fractures Deficit of the extensor mechanism with implantation of non-articulating spacer

ICM: International Consensus Meeting; PJI: periprosthetic joint infection

### Ethical approval

for this study was obtained from the Department of Infectious Diseases of the D. Cotugno Hospital. The study was conducted in accordance with national and institutional standards and in accordance with the principles of the Declaration of Helsinki. The patients provided informed consent before they were included in the study. The characteristics of patients are reported in Table 2.

**Table 2:** Characteristics of the patients (*n* = 108)

Patients data	Mean ± SD (range) or N (%)
Age	68.9 ± 9.7 (45–87)
Sex	
Female	43 (39.8)
Male	65 (60.2)
Body mass index	28.5 ± 4.7 (19.7–45.6)
Educational level	
Primary School	42 (38.9)
Secondary School	45 (41.7)
High School	13 (12.0)
Graduation	8 (7.4)
Cigarette smoking	
Non-smokers	78 (72.2)
Regular smokers	30 (27.8)
Charlson Comorbidity Index	4.5 ± 4.9 (0–28)
Previous surgeries	
1	69 (63.9)
2	32 (29.6)
3	5 (4.6)
4	2 (1.9)
Microbiological culture	
Positive	48 (44.4)
Negative	60 (55.6)
Isolated bacterial strains	
MS-CONS	11 (22.9)
MR-CONS	9 (18.8)
MRSA	7 (14.6)
MSSA	5 (10.4)
Gram-negative bacilli	5 (10.4)
*Enterococcus faecalis*	3 (6.3)
Others	8 (16.7)

SD=Standard Deviation; MS-CONS= Methicillin-sensitive coagulase-negative *Staphylococcus*; MR-CONS= Methicillin-resistant coagulase-negative *Staphylococcus*; MRSA= Methicillin-resistant *Staphylococcus aureus;* MSSA= Methicillin-sensitive *Staphylococcus aureus*

### Treatment Regimen

Intravenous antibiotic treatment was scheduled for 2 weeks after implant removal, followed by targeted oral therapy for 6 weeks when feasible [14]. When the synovial fluid (SF) culture results were negative, empiric antibiotic therapy active against methicillin-resistant staphylococci was administered until the results of the cultures of periprosthetic tissue or implant sonication fluid be-came available. After completing the antibiotic course, reimplantation was planned while still receiving antibiotic treatment (continuous antibiotic therapy) [15]. Reimplantation was scheduled for patients whose C-reactive protein (CRP) level and erythrocyte sedimentation rate (ESR) remained normal or had decreased and who had no local signs or symptoms of infection. All the patients meeting these criteria, but only sixty-five proceeded to second-stage reimplantation. In contrast, in 43 patients the spacer was retained indefinitely. The decision was shared with patients considering age, comorbidity and previous surgeries at same site. The median time interval from the prosthesis removal to reimplantation was 8 weeks (range, 8 to 9 weeks).

We defined the absence of PJI as the disappearance of all clinical, microbiological, and radiographic evidence of PJI during the 96-week follow-up period after the discontinuation of antibiotics. By this definition, 91.6% (99/108) of patients were infection-free, while 8.3% (9/108) experienced recurrence. For the 43 patients in which the spacer was retained indefinitely, two patients were lost to follow-up, and one patient died from causes unrelated to the index procedure. Five revisions occurring out of the initial cohort of 40 cases by the 42-month follow-up for mechanical failure (joint instability or patients’ dissatisfaction).

### Data collection and patients’ assessment

Patients’ personal and medical data were collected, including a full physical examination before surgery. QoL and joint function were assessed using the Eq. 5D5L [16], the WOMAC Questionnaire [17], and the Knee Society Score (KSS) [18]. All the outcomes were assessed again before planned reimplantation. Comorbidities were evaluated using the Charlson Comorbidity Index [19]. All these instruments are explained in detail in the appendix.

### Statistical analysis

Normality of continuous variables and change scores was assessed using the Shapiro–Wilk test. Statistical analyses included a two-sample t-test and ANOVA to assess cross-sectional differences, with a Bonferroni test for multiple comparisons. A Pearson’s coefficient was used to evaluate correlations between outcomes. Separate models of stepwise multiple linear regression analysis were used to identify predictors of QoL and knee function before and after spacer implantation. The outcomes included in the models were the Eq. 5D5L index, EQ-VAS, EQ-5D-5 L dimensions, WOMAC index, KSS-knee (KSS-K), and KSS-function (KSS-F). Patient-reported outcome measures were collected preoperatively, before the first-stage surgery. Outcomes were reassessed at a median of 8 weeks (range, 8–9 weeks) after spacer implantation in all patients. In patients who subsequently underwent second-stage revision, PROMs were obtained before reimplantation at the same time point. In patients who retained the spacer, PROMs were collected at the same median interval. The explanatory variables and possible confounders included in the models were age (continuous), gender (categorical), BMI (body mass index) (continuous), cigarette smoking (categorical), CCI (Charlson Comorbidity Index) (continuous), education level (discrete, 4-step scale), number of previous surgeries (continuous), previous revision surgeries (categorical), and positive culture result (categorical). In the separate models testing predictors of PROMs after spacer implantation, the baseline value of the PROM under examination was also tested as an explanatory variable. Before constructing the single models of stepwise multiple linear regression analysis, an age-adjusted univariate linear regression analysis was performed by testing each individual explanatory variable. Explanatory variables were included in the multiple regression models if a trend toward an association (i.e. *p* < 0.10) with the outcome of interest was found in the univariate analysis. The minimum clinically important difference (MCID) for each outcome, using a distribution-based method [20] was calculated to assess whether the degree of change in general health and knee function obtained with surgery constituted a positive outcome. With this method, the discrimination threshold for clinically meaningful changes in a single outcome is half a standard deviation of the baseline value of the same outcome. A receiver operating characteristic (ROC) curve analysis was used to determine an optimal threshold value for the PROMs evaluated separately. The area under the curve (AUC) of each ROC analysis indicated the predictive validity of this binary classifier test for predicting the likelihood that a patient would achieve the MCID for the outcome. The calculated threshold value specified a preoperative score best able to predict the likelihood of a patient experiencing a MCID after implantation of the spacer. A post-hoc power analysis was performed based on the EQ-5D-5 L change score. The achieved statistical power exceeded 90% (α = 0.05), given the observed effect size and the sample size (*n* = 108). All statistical tests were conducted using SPSS software (SPSS, Inc., Chicago, IL, USA), with significance set at *p* < 0.05.

## Results

### EQ-5D-5 L

Table 3 presents the EQ-5D-5 L scores. No significant age-related differences were found in the EQ-5D-5 L Index or EQ-VAS before and after surgery, except for a higher preoperative EQ-VAS score in the youngest group (*p* = 0.024). All age groups showed significant improvement in QoL after implantation of the spacer. The mean change exceeded MCID thresholds for post-operative improvement calculated with a distribution-based method and the MCIDs previously reported after TKA [21,22]. Specifically, with surgery, 84 (77.8%) and 91 (84.3%) patients achieved MCID for the EQ-5D-5 L index and EQ-VAS, respectively. Despite the significant postoperative improvement, the mean EQ-5D-5 L and EQ-VAS of patients with knee spacer still compared negatively with normative values, and these differences constantly exceeded MCIDs [21,22].

Table 4 shows the responses of the EQ-5D-5 L dimensions at baseline and before reimplantation. Preoperatively, the most impaired dimensions of the EQ-5D-5 L were pain/discomfort, usual activities, and mobility. Surgery resulted in significant improvement (*p* < 0.001) for all five dimensions, with the greatest improvement being observed for pain/discomfort. Before reimplantation, 68.6% to 84.2% of the patients reported ‘mild’ or ‘no’ problems, depending on the EQ-5D-5 L dimension.

**Table 3:** EQ-5D-5L Index and EQ-VAS results stratified by 4 age groups in patients (PTS) in comparison with age-matched population norms (Norms) [45]

		EQ‑5D‑5 L Index Value						EQ-VAS						
		PTS					Norms	PTS					Norms	
	N	Preoperative	Postoperative	p-value	Variation	95% CI		Preoperative	Postoperative	p-value	Variation	95% CI		
All patients	108	0.22 ± 0.38	0.72 ± 0.31	< 0.001	0.50 ± 0.44	0.41–0.58	0.93 ± 0.11	30.8 ± 18.9	66.4 ± 24.7	< 0.001	35.6 ± 30.1	29.8–41.3	81.8 ± 13.5	
Age 45–54	10	0.23 ± 0.36	0.65 ± 0.39	0.003	0.42 ± 0.33	0.19–0.66	0.93 ± 0.09	48.0 ± 22.0	70.0 ± 24.9	0.068	22.0 ± 33.6	-2.0-46.0	82.4 ± 12.9	
Age 55–64	23	0.26 ± 0.43	0.72 ± 0.31	< 0.001	0.53 ± 0.46	0.33–0.73	0.91 ± 0.14	30.0 ± 18.6	71.3 ± 23.8	< 0.001	41.3 ± 27.0	29.6–53.0	79.6 ± 15.3	
Age 65–74	42	0.21 ± 0.37	0.69 ± 0.32	< 0.001	0.48 ± 0.53	0.32–0.65	0.91 ± 0.15	28.8 ± 18.0	62.6 ± 26.2	< 0.001	33.8 ± 31.4	24.0-43.6	78.2 ± 14.8	
Age ≥ 75	33	0.21 ± 0.36	0.72 ± 0.27	< 0.001	0.51 ± 0.35	0.39–0.64	0.91 ± 0.13	28.8 ± 17.3	66.7 ± 23.7	< 0.001	37.9 ± 29.1	27.6–48.2	75.1 ± 16.4	

**Table 4:** EQ-5D-5L dimension at baseline and before planned reimplantation

Dimension	Level of severity										
	No problems (1)		Slight problems (2)		Moderate problems (3)		Severe problems (4)		Extreme/Unable (5)		Mean ± SD
Preoperative											
Mobility	4	3.7%	17	15.7%	50	46.3%	19	17.6%	18	16.7%	3.28 ± 1.04
Self-care	18	16.7%	15	13.9%	42	38.9%	22	20.4%	11	10.2%	2.94 ± 1.20
Usual activities	11	10.2%	9	8.3%	44	40.7%	19	17.6%	25	23.1%	3.35 ± 1.22
Pain/discomfort	1	0.9%	12	11.1%	46	42.6%	24	22.2%	25	23.1%	3.56 ± 1.00
Anxiety/depression	32	29.6%	26	24.1%	20	18.5%	12	11.1%	18	16.7%	2.61 ± 1.44
Postoperative											
Mobility	45	41.7%	29	26.9%	28	25.9%	4	3.7%	2	1.9%	1.97 ± 1.00
Self-care	68	63.0%	18	16.7%	16	14.8%	2	1.9%	4	3.7%	1.67 ± 1.04
Usual activities	54	50.0%	30	27.8%	12	11.1%	8	7.4%	4	3.7%	1.87 ± 1.11
Pain/discomfort	52	48.1%	29	26.9%	15	13.9%	9	8.3%	3	2.8%	1.91 ± 1.10
Anxiety/depression	71	65.7%	20	18.5%	11	10.2%	1	0.9%	5	4.6%	1.60 ± 1.03

### KSS and WOMAC

Table 5 shows the results of the KSS and WOMAC indices in the patients stratified by 4 age groups. No significant differences were found between the different age groups in these scores before the operation. Before the planned reimplantation, there was significant improvement in pain and knee function scores in all age groups. The improvement achieved with surgery exceeded MCIDs for all the outcomes calculated with a distribution-based method and previously reported MCIDs for improvement after TKA [23–25]. In detail, with surgery, 84 (77.8%), 67 (62%), and 84 (77.8%) patients achieved MCID for the KSSK, KSSF, and WOMAC Index, respectively. The mean post-operative KSSF was significantly better in the younger age group than in the older age group.

**Table 5:** Knee society score (KSS) and WOMAC score results stratified by 4 age groups in patients (PTS)

		N	Preoperative	Postoperative	*p-value*	Variation	95% CI
KSS Knee Score	All patients	108	40.54 ± 17.45	62.97 ± 18.10	0.030	22.44 ± 22.36	18.17–26.70
	Age 45–54	10	50.70 ± 14.85	67.50 ± 17.62	0.022	16.80 ± 19.13	3.12–30.48
	Age 55–64	23	42.26 ± 18.15	67.78 ± 13.80	< 0.001	25.52 ± 21.95	16.03–35.01
	Age 65–74	42	40.31 ± 15.13	59.43 ± 20.02	< 0.001	19.12 ± 23.23	11.88–26.36
	Age ≥ 75	33	36.55 ± 19.63	62.76 ± 18.01	< 0.001	26.21 ± 22.37	18.28–34.14
	*p-value*		0.147	0.279		0.406	
KSS Function Score	All patients	108	29.49 ± 25.68	51.20 ± 26.68	< 0.001	21.71 ± 30.02	15.99–27.44
	Age 45–54	10	41.50 ± 9.73	64.50 ± 26.61	0.028	23.00 ± 27.91	3.04–42.96
	Age 55–64	23	29.78 ± 28.06	54.57 ± 26.41	0.001	24.78 ± 29.29	12.12–37.45
	Age 65–74	42	30.60 ± 26.46	55.24 ± 24.47	< 0.001	24.64 ± 33.49	14.22–35.07
	Age ≥ 75	33	24.24 ± 25.77	39.70 ± 26.66	0.002	15.45 ± 26.67	6.00-24.91
	*p-value*		0.305	0.017		0.559	
WOMAC Score	All patients	108	47.83 ± 18.18	22.34 ± 19.03	< 0.001	25.49 ± 23.61	20.99–29.99
	Age 45–54	10	45.90 ± 15.72	20.60 ± 20.18	0.005	25.30 ± 21.60	9.85–40.75
	Age 55–64	23	46.65 ± 19.37	18.48 ± 16.63	< 0.001	28.17 ± 18.09	20.35-36.00
	Age 65–74	42	48.02 ± 18.23	25.26 ± 20.96	< 0.001	22.76 ± 25.03	14.96–30.56
	Age ≥ 75	33	49.00 ± 18.67	21.85 ± 17.89	< 0.001	27.15 ± 26.22	17.86–36.45
	*p-value*		0.952	0.569		0.801	

### Gender differences

At baseline, men showed more impaired functional status and QoL than women. Indeed, the preoperative mean EQ-5D-5 L index, EQ-VAS, KSSF, and WOMAC index showed significant sex-related differences. Following the spacer implantation, there was significant postoperative improvement in all parameters in both sexes, but a gender difference persisted in the KSSF and the WOMAC index, which maintained worse results in males than in females. Considering the five dimensions of the EQ-5D-5 L questionnaire, mean preoperative usual activity was significantly more impaired in men. No other gender differences in the dimensions of EQ-5D-5 L were found. Data on gender differences are detailed in the appendix.

### Correlation and regression analysis

Preoperatively, a weak to moderate correlation (*p* ≤ 0.002) was found between QoL and knee function indices, with the strongest relationship between WOMAC and KSSF (c = -0.561; *p* < 0.001). Postoperatively, this correlation was stronger, with WOMAC and EQ-VAS showing the highest association (c = -0.791; *p* < 0.001). Multiple linear regression identified female gender as a positive determinant of baseline EQ-5D-5 L Index, EQ-VAS, EQ-5D Usual Activities, WOMAC Index, and KSSF. The gender differences accounted for 3.8% to 10.9% of the variance in the models. The baseline QoL and knee function inversely correlated with the postoperative outcome, and the preoperative status was the strongest predictor of all scores (Table 6).

**Table 6:** Effect of the baseline level on postoperative improvement of the same outcome (Multiple linear regression analysis)

Outcome	C	*P*	*R*^2^%
EQ-5D-5 L Index	-0.847	< 0.001	53.8
EQ-VAS	-0.914	< 0.001	32.6
EQ-5D-5 L Dimension			
Mobility	0.941	< 0.001	49.4
Self-care	0.895	< 0.001	52.1
Usual activities	0.893	< 0.001	49.3
Pain/discomfort	0.910	< 0.001	40.8
Anxiety/depression	0.809	< 0.001	57.8
KSSK	-0.783	< 0.001	37.3
KSSF	-0.682	< 0.001	30.3
WOMAC Index	0.790	< 0.001	37.5

**Fig. 2:**
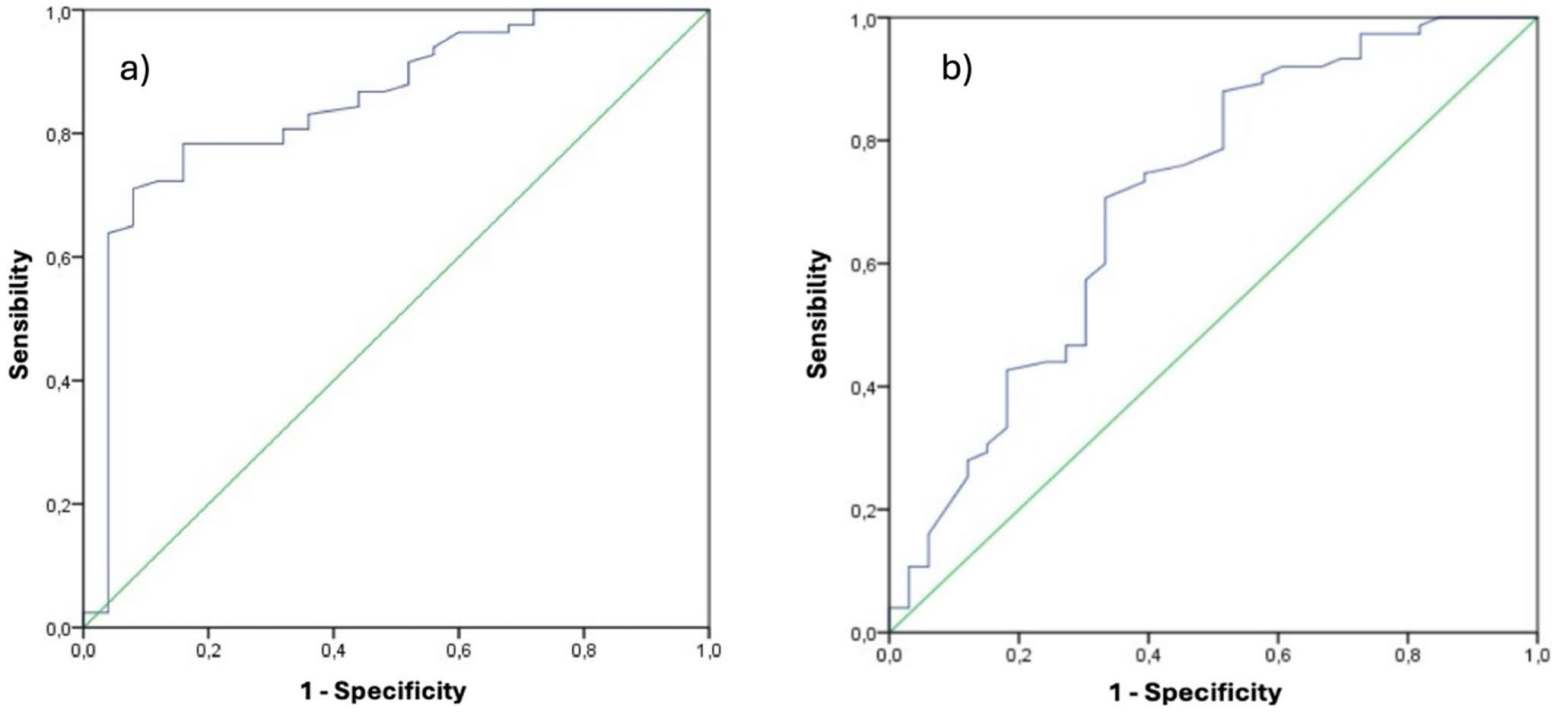
Representation of AUC of the Eq. 5D5L index (**a**) and WOMAC Index (**b**)

In other words, the poorer the patients’ baseline conditions, the greater the post-operative improvement. Using ROC curves, an acceptable predictive threshold baseline value was identified for the Eq. 5D5L and WOMAC Indexes. The ROC curve analysis established predictive thresholds: an EQ-5D-5 L index ≤ 0.44 (sensitivity 0.78, specificity 0.84) and a WOMAC index ≥ 42.5 (sensitivity 0.71, specificity 0.67) predicted MCID achievement. The AUC values were 0.85 (EQ-5D-5 L) and 0.71 (WOMAC) (Fig. 2).

## Discussion

This study investigated functional and quality-of-life (QoL) outcomes in patients with chronic periprosthetic knee infection treated with a metal-on-polyethylene articulating spacer as part of a planned two-stage revision. The main findings were: (1) baseline QoL and knee function were markedly impaired; (2) significant improvements were observed after spacer implantation, with changes exceeding established and distribution-based minimum clinically important difference (MCID) thresholds for multiple outcome measures; (3) despite these improvements, postoperative scores remained below age-matched population norms; and (4) poorer preoperative status predicted greater postoperative gains, with preoperative EQ-5D-5 L and WOMAC thresholds providing clinically useful benchmarks for anticipating meaningful improvement.

Multiple studies have shown that patients with PJIs have reduced QoL, decreased ability to carry out everyday activities, and impaired joint functionality [26–28]. Although two-stage exchange has traditionally been considered the gold-standard technique for treating PJI [29], it significantly impacts patients’ QoL and functionality [30]. Using a metal-on-poly spacer has been shown to provide acceptable function [31,32] and some frail, low-demand individuals or those with medical comorbidities that preclude the second-stage surgery may be satisfied with the spacer and avoid a second surgery. Also in this study, the implantation of the spacer led to marked improvement in QoL and knee function, which occurred irrespective of age and was greater than MCIDs calculated using a distribution-based method and those recorded in patients undergoing TKA for Eq. 5D5L and EQ-VAS index [23,24], for the KSSK and KSSF^25^, and for the WOMAC index [24,25]. Despite the significant postoperative improvement, the mean EQ-5D-5 L and EQ-VAS values in spacer patients were lower than normative values, with differences consistently exceeding MCIDs [21,22]. Comparisons with previous studies are challenging due to limited published data, but some reports indicate similar outcomes. One study found a lower EQ-5D index before reimplantation in two-stage patients, which improved to 0.71 three months after revision [29], a result similar to the current study. Budin et al.^33^ reported mean EQ-5D-5 L scores of 0.634 and 0.671 in one- and two-stage knee revisions after 54.5 months, alike to our results. In this study, pain/discomfort was the most impaired EQ-5D-5 L dimension at baseline but it showed the greatest postoperative improvement. Conversely, anxiety/depression was the least affected. No previous studies have examined EQ-5D-5 L dimensions in PJI patients who had undergone placement of a metal-on-polyethylene spacers, but similar variations have been observed in TKA patients by Conner-Spady et al. [21]. In terms of joint-specific parameters, the reported KSSK (72.7–91.7) and KSSF (48.3–88.3) in PJI patients with prosthetic articulating spacers were slightly better than ours [34–36], whereas the WOMAC index in our study compares favorably with Song et al. findings [37]. In the current study, the postoperative improvements in WOMAC and KSSF exceeded treatment success thresholds for TKA [38], whereas KSSK did not, likely due to differences in the functional aspects assessed by each questionnaire. We also observed sex-related differences, with females demonstrating better preoperative QoL and knee function, particularly in usual activities. These differences persisted postoperatively, with men showing greater impairment. While previous studies indicate men are more prone to PJI [39], others report lower QoL in women before primary TKA [40,41]. This discrepancy may be due to variations in patient populations, diagnoses, or unaccounted confounding factors. Furthermore, Bischof et al. [41] found that the EQ-5D self-care dimension is positively associated with female gender and concluded that the different associations of gender with individual dimensions of the EQ-5D-5 L after TKA need to be considered and interpreted in detail. Finally, it was reported that women obtain higher change scores after the intervention and have a faster recovery 3 months after TKA, which is an interval similar to the present study [40].

This study found that baseline EQ-5D-5 L Index, KSS and WOMAC scores were independent inverse predictors of achieving the MCID after spacer implantation. Patients with poorer preoperative status were more likely to experience meaningful postoperative improvements. To our knowledge, this is the first study to demonstrate such findings specifically in PJI patients treated with an articulating spacer. Similar trends have been observed in TKA patients, as Berliner et al. demonstrated that higher preoperative KOOS and SF-12 PCS scores reduced the likelihood of meaningful improvement [42]. Likewise, McCabe et al. found that a lower preoperative EQ-5D-5 L index predicted MCID achievement after TKA [43]. We also identified predictive thresholds for postoperative success: an EQ-5D-5 L index ≤ 0.44 and a WOMAC index ≥ 42.5 increased the likelihood of obtaining meaningful functional improvement. The identification of these thresholds associated with MCID achievement provides a practical tool for clinicians considering interim or definitive retention of an articulating spacer (1.5 stage), especially in elderly or comorbid patients for whom the risks of a second major surgery may outweigh potential benefits. This study has limitations, including its single-centre design, the absence of a control group, the reliance on MCID thresholds, and lack in internal validation of the predictive models. MCID thresholds were derived from osteoarthritis-related TKA populations and the achievement of MCID with surgery for all outcomes was calculated using a distribution-based method, which has inherent limitations. Indeed, it does not reflect the change perceived by the patient and may overestimate clinical relevance in severely compromised patients. Therefore, these results are exploratory in nature. Internal validation of predictive models, such as bootstrap resampling, was not performed in the study, raising concerns about overfitting and limiting the clinical utility of the baseline cut-off values for EQ-5D-5 L and WOMAC identified in the ROC curve analysis. Lastly, the results reflect short-term outcomes prior to reimplantation, and infection eradication data were not the primary focus. Nevertheless, the study has several strengths: it is based on a relatively large prospectively collected cohort, uses validated PROMs, applies robust statistical analyses, and—importantly—identifies clinically relevant preoperative thresholds predictive of meaningful improvement. These findings provide novel and practical insights that can guide patient selection and inform clinical decision-making, particularly in the context of the 1.5-stage approach [32,44].

## Conclusion

Metal-on-polyethylene articulating spacers used during stage-1 surgery for chronic knee periprosthetic joint infection are associated with meaningful short-term improvements in function and QoL in patients with chronic knee PJI awaiting second-stage revision. Patients with poorer baseline status appear most likely to achieve clinically meaningful benefits. While these findings may support selective indication to leave the spacer in place indefinitely (1.5 approach), avoiding further surgeries, confirmation through longer-term, comparative, and cost-effectiveness studies is needed before widespread adoption.

## Supplementary Information

Below is the link to the electronic supplementary material.

Supplementary Material 1.

## Data Availability

The datasets generated and analyzed during the current study are available from the corresponding author upon reasonable request.
