# Supplementary material for: Functional and quality-of-life outcomes after metal-on-polyethylene articulating spacer implantation for periprosthetic knee infection: a retrospective evaluation of a prospectively collected cohort
Source: Arch Orthop Trauma Surg. 2026 Apr 17;146(1):142. doi: 10.1007/s00402-026-06260-0 (PMC13090179; doi:10.1007/s00402-026-06260-0)
Supplement: Supplementary file 1 — Supplementary Material 1. [file 402_2026_6260_MOESM1_ESM.docx]

**Appendix**

**Instruments for patients’ assessment**

| **Instrument/Index** | **Description** | **Scoring/Levels** | **Interpretation** |
| --- | --- | --- | --- |
| **EQ-5D-5L** | Standardized measure of Health-Related Quality of Life (HRQoL) with 5 dimensions: mobility, self-care, usual activities, pain/discomfort, and anxiety/depression. | 5 levels for each dimension: No problems, Slight problems, Moderate problems, Severe problems, Extreme problems. | Higher levels indicate worse health status in each dimension. |
| **WOMAC (Western Ontario and McMaster Universities Osteoarthritis Index)** | Self-administered measure assessing pain, stiffness, and physical function. | Total score on a 0–100 scale (adjusted). | Lower scores indicate less pain/stiffness and better function. |
| **KSS (Knee Society Score)** | Clinician-administered tool with two components: Knee Score (KSSK) and Function Score (KSSF). | KSSK: 0–100 points for range of motion, stability, and pain. KSSF: 0–100 points for walking and stair climbing (deductions for walking aid use). | Higher scores indicate better knee status and function. |
| **Charlson Comorbidity Index (CCI)** | Assesses the burden of chronic illnesses, predicting mortality risk based on 19 health conditions. | Numeric score (0 = no comorbidities). | Higher scores indicate a greater burden of chronic illness and higher predicted mortality. |

**Data on gender differences**

| **Parameter** | **Preoperative (Men)** | **Preoperative (Women)** | **p-value (Pre-op)** | **Postoperative (Men)** | **Postoperative (Women)** | **p-value (Post-op)** |
| --- | --- | --- | --- | --- | --- | --- |
| **EQ-5D-5L Index** | 0.76 ± 0.31 | 0.68 ± 0.30 | 0.024 | Significant improvement (both) | Significant improvement (both) | ≤ 0.001 |
| **EQ-VAS** | 37.91 ± 18.33 | 26.15 ± 17.83 | 0.001 | Significant improvement (both) | Significant improvement (both) | ≤ 0.001 |
| **KSSF (Function Score)** | 22.62 ± 25.44 | 39.88 ± 22.59 | <0.001 | 46.77 ± 26.06 | 57.91 ± 26.46 | 0.033 |
| **WOMAC Index** | 51.60 ± 18.23 | 42.14 ± 16.73 | 0.007 | 25.32 ± 19.90 | 17.84 ± 16.86 | 0.038 |
| **EQ-5D-5L Usual Activities** | 3.55 ± 1.25 | 3.05 ± 1.11 | 0.030 | Significant improvement (both) | Significant improvement (both) | ≤ 0.001 |
| **EQ-5D-5L Anxiety/Depression** | No significant difference | No significant difference | — | 1.78 ± 1.10 | 1.33 ± 0.87 | 0.023 |
